# Relationship between wave-like auroral arcs and Pi2 disturbances in plasma sheet prior to substorm onset

**DOI:** 10.1186/s40623-015-0334-8

**Published:** 2015-10-13

**Authors:** Tzu-Fang Chang, Chio-Zong Cheng

**Affiliations:** 1 Institute of Space and Plasma Sciences, National Cheng Kung University, Tainan, Taiwan; 2 Department of Physics, National Cheng Kung University, Tainan, Taiwan; 3 Plasma and Space Science Center, National Cheng Kung University, Tainan, Taiwan; 4 Department of Advanced Energy, University of Tokyo, Tokyo, Japan

## Abstract

Wave-like substorm arc features in the aurora and Pi2 magnetic disturbances observed in the near-Earth plasma sheet are frequently, and sometimes simultaneously, observed around the substorm onset time. We perform statistical analyses of the THEMIS ASI auroral observations that show wave-like bright spot structure along the arc prior to substorm onset. The azimuthal mode number values of the wave-like substorm arcs are found to be in the range of ~100–240 and decrease with increasing geomagnetic latitude of the substorm auroral arc location. We suggest that the azimuthal mode number is likely related to the ion gyroradius and azimuthal wave number. We also perform correlation study of the pre-onset wave-like substorm arc features and Pi2 magnetic disturbances for substorm dipolarization events observed by THEMIS satellites during 2008–2009. The wave-like arc brightness structures on the substorm auroral arcs tend to move azimuthally westward, but with a few exceptions of eastward movement, during tens of seconds prior to the substorm onset. The movement of the wave-like arc brightness structure is linearly correlated with the phase velocity of the Pi2 δ*B*_*y*_ disturbances in the near-Earth plasma sheet region. The result suggests that the Pi2 transverse δ*B*_*y*_ disturbances are related to the intensifying wave-like substorm onset arcs. One plausible explanation of the observations is the kinetic ballooning instability, which has high azimuthal mode number due to the ion gyroradius effect and finite parallel electric field that accelerates electrons into the ionosphere to produce the wave-like arc structure.

## Background

The launch of the Time History of Events and Macroscale Interactions during Substorms (THEMIS) mission (Angelopoulos 2008) has provided researchers with data of simultaneous ground, ionospheric, and magnetospheric observations to investigate the substorm onset phenomena which typically manifest within the crucial tens of seconds around substorm onset time.

One of the important phenomena of the substorm auroral arcs observed by THEMIS Ground-Based Observatory (GBO), which consists of a network of all-sky imagers (ASIs), magnetometers, etc. (Mende et al. 2008), is that the substorm onset arcs are characterized by wave-like bright spot structure prior to substorm onset (e.g., Donovan et al. 2006; Liang et al. 2007; Sakaguchi et al. 2009; Uritsky et al. 2009; Rae et al. 2010; Chang et al. 2012), and the intensity of substorm onset arc is found to grow exponentially during the pre-onset period of time (e.g., Voronkov et al. 1999, 2003; Donovan et al. 2008; Liang et al. 2008; Rae et al. 2010; Chang et al. 2012). It is generally suggested that the wave-like arc brightness structure is related to some instability excited in the inner plasma sheet. Thus, the azimuthal structure and luminosity growth of substorm onset arcs would impose important constraints to the near-Earth instabilities for understanding the substorm onset mechanism.

In the magnetosphere, ultra low frequency (ULF) disturbances in the Pi2 (40–150 s period) and Pi1 (1–40 s period) frequency ranges have also been investigated in relation to substorm onset. In many substorm events, Pi2 disturbances appear prior to the magnetic field dipolarization onset in the plasma sheet and Pi1 disturbances are commonly observed at or after the substorm onset (e.g., Takahashi et al. 1987, Roux et al. 1991; Cheng and Lui 1998; Shiokawa et al. 2005). Before the launch of THEMIS spacecraft quintet, most in situ substorm observations were performed by the AMPTE and Geotail satellites. Based on the AMPTE/CCE data, Cheng and Lui (1998) identified a ULF instability with a wave period of about 60–100 s (in the Pi2 range), which is excited about 2–3 min before the current disruption starts. Geotail satellite has also observed many substorm dipolarization events with Pi2 wave activities in the plasma sheet (e.g., Shiokawa et al. 2005; Saito et al. 2008). With THEMIS satellites, both Pi1 and Pi2 disturbances were detected in almost all observed magnetospheric substorm dipolarization events (e.g., Lui et al. 2008; Uritsky et al. 2009; Keiling 2012).

Theories of instabilities (e.g., Lui et al. 1991; Roux et al. 1991; Yoon et al. 1994, 1996; Lui 1996; Zhu and Winglee 1996; Liu 1997; Pu et al. 1997; Voronkov et al. 1997; Cheng and Lui 1998; Perraut et al. 2000; Cheng 2004) have been proposed to explain the underlying mechanisms that initiate the onset of substorm expansion. To investigate the relationship between wave-like auroral arcs and instabilities, kinetic ballooning instability has drawn much attention. Cheng and Lui (1998) studied the kinetic ballooning instability by including trapped electron dynamics and finite ion gyroradius effects to explain the Pi2 disturbance excited prior to the initiation of current disruption. The kinetic ballooning instability calculations have shown that the most unstable modes occur in the strong cross-tail current region that maps to the ionosphere to form a substorm auroral arc formation similar to the ideal MHD calculation results (Cheng and Zaharia 2004). Saito et al. 2008 reported substorm events in which GEOTAIL observes the ballooning mode perturbations initiated ~2 min prior to the dipolarization onset. Panov et al. (2012) showed the THEMIS spacecraft observations of field and plasma oscillations prior to breakup, which confirm the predictions of the kinetic ballooning/interchange instability. Chang et al. (2012) and Xing et al. (2013) also examined the role of the kinetic ballooning instability in a substorm event and explain several observational features. Because the kinetic ballooning instability has finite parallel electric field and high azimuthal mode number, it is suggested that the excitation of kinetic ballooning instability may be responsible for producing the pre-onset wave-like arc brightness structure in the ionosphere during substorm.

Even though various ionospheric and magnetospheric substorm phenomena have been explored in many studies, there have been relatively few efforts in attempting to investigate the correlation between the substorm auroral activities and the magnetospheric features associated with the magnetotail dipolarization process. Moreover, it is critically important to compare the observational features of the pre-onset phenomena with the theoretical predictions. Xing et al. (2013) showed a case study to identify auroral wave structures and examine plasma sheet dynamics using observations of THEMIS multi-spacecraft conjunction. In this paper, we perform statistical studies of the features of the wave-likesubstorm auroral arcs and magnetic disturbances in the plasma sheet by examining substorm dipolarization events observed by THEMIS satellites during 2008–2009 which are defined in the substorm timing table provided by UCLA (http://www.igpp.ucla.edu/themis/events/). We start by presenting the detailed study of the 26 Feb. 2008 substorm event. Then, we present the correlation between the auroral arc wave-like structure and the Pi2 disturbance activity observed by THEMIS spacecraft for these substorm events. Our result shows that prior to the substorm onset the moving velocity of the wave-like arc bright spot structure is proportional to the phase velocity of the Pi2 δ*B*_*y*_ disturbances in the plasma sheet. We also show that the azimuthal mode number of the substorm onset arc wave-like structure is similar to the azimuthal mode number of the Pi2 δ*B*_*y*_ disturbances in the plasma sheet. Moreover, we find that the azimuthal mode number of the substorm onset arc wave-like structure is smaller for substorm arcs located at higher magnetic latitude (MLAT). Finally, we discuss the role of the kinetic ballooning instability (e.g., Cheng 1982a, 1982b, 2004; Cheng and Lui 1998; Cheng and Gorelenkov 2004) as a plausible candidate for substorm mechanism in understanding qualitatively the analysis results of these simultaneous observations of the ionospheric and magnetospheric substorm phenomena. It should be noted that no theoretical calculation of kinetic ballooning instability has been performed to compare with the observation quantitatively. There could be other mechanism which is yet to be explored to explain the analysis results of observations presented in this paper.

## Methods

### The 26 Feb. 2008 substorm event

We first present the ionospheric and magnetospheric features of a specific substorm event with the auroral onset time at ~0404 UT on 26 February 2008. In particular, we find that within ~60 s prior to the substorm onset the auroral wave-like arc structure moves westward with average moving velocity about the same as the phase velocity of the westward propagating Pi2 δ*B*_*y*_ disturbance observed by the THEMIS D and THEMIS E (Th D and Th E) satellites if the Pi2 disturbance activity is mapped by using the T96 model (Tsyganenko 1995) to the ionosphere. This substorm event was previously examined by Pu et al. (2010) in relation with magnetic reconnection in the magnetotail and by Ogasawara et al. (2011) on the relation between the auroral features and magnetispheric plasma flow and *B*_*z*_ dipolarization which occurred just after the auroral expansion onset. Here, we focus on the dynamics of the wave-like substorm arc prior to the substorm onset and its relationship with the corresponding Pi2 disturbance activity in the near-Earth plasma sheet.

#### Auroral arc features of the 26 Feb. 2008 substorm

**Fig. 1:**
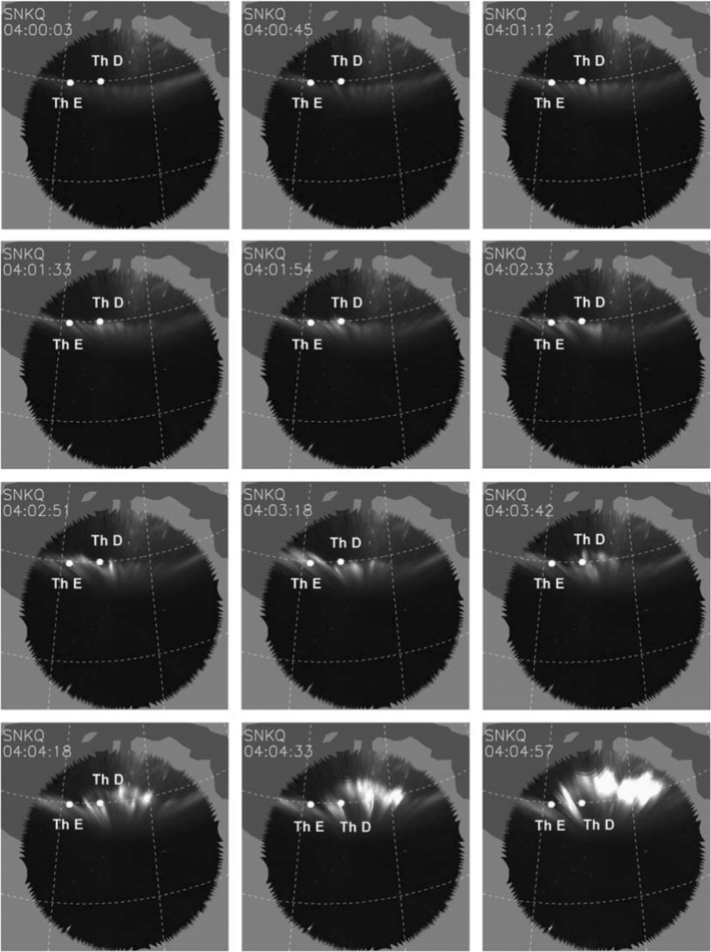
The sequence of SNKQ ASI auroral images shows the evolution of substorm auroral expansion with substorm onset at ~0404:30 UT on 26 Feb. 2008. The *white dots* in these images indicate the fieldline footprints of the Th D and Th E spacecraft locations mapped by using the T96 model

The auroral activities of the 26 Feb. 2008 substorm event were observed by the ground-based THEMIS ASIs located at Sanikiluaq (SNKQ). The time evolution of the auroral substorm arc images was captured in the SNKQ ASI field-of-view (FOV) and is shown in Fig. 1. The white dots in Fig. 1 indicate the fieldline footprints of the Th D and Th E spacecraft in the ionosphere mapped by using the Tsyganenko 96 (T96) model before substorm onset when the magnetic field perturbation is very weak. At ~0403 UT, the Th D and Th E spacecraft are located at (−10.6, 4.2, −1.9) R_E_ and (−9.8, 4.8, −1.6) R_E_ in the GSM coordinates, respectively. The separation distance between Th D and Th E spacecraft is (Δ*x*, Δ*y*, Δ*z*) = (0.8, 0.6, 0.3) R_E_, and the azimuthal separation distance is Δ*R*~1 R_E_. The footprints of Th D and Th E spacecraft are located at ~(279° LON, 70° MLAT) and (276° LON, 70° MLAT), respectively, which are roughly on the substorm initiation arc at 0404 UT (just prior to the auroral substorm onset time), and their separation distance is ~156 km.

**Fig. 2:**
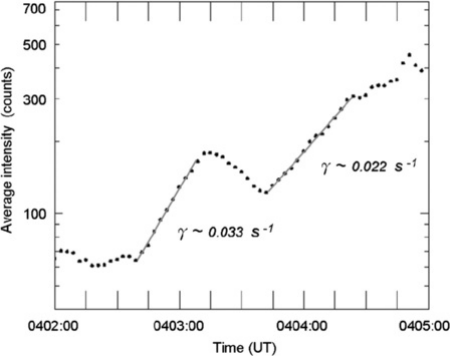
Temporal evolution of the average substorm auroral arc intensity (in unit of SNKQ ASI detector). The average auroral arc intensity is found to grow by two exponential stages

Initially (at ~0400 UT) a relatively stable pre-existing arc was located at ~69.5° MLAT and extends azimuthally in the FOVs of GILL and SNKQ. At ~0401:00 UT, the pre-existing arc gradually brightened and evolved into wave-like bright spot structure along the arc. The wave-like substorm arc continued to brighten, and from ~0402:40 UT the average auroral arc intensity (in unit of SNKQ ASI detector) intensified with two exponentially growing stages as shown in Fig. 2. Note that in determining the wave-like substorm arc intensity we have separated the arc intensity from other regions. During 0402:40–0403:10 UT, the average arc intensity grew exponentially with a growth rate of ~3.3 × 10^−2^ s^−1^ and then decreased. Then, from 0403:40 to 0404:30 UT, the average arc intensity intensified again with an exponential growth rate of ~2.2 × 10^−2^ s^−1^. At ~0404:30 UT, the brightening wave-like substorm arc started to be rapidly expand both poleward and azimuthally in the SNKQ FOV. We regard 0404:30 UT as the expansion onset of this auroral substorm event.

**Fig. 3:**
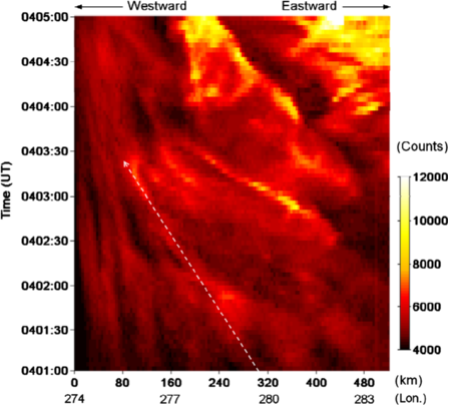
Wave-like substorm auroral arc intensity as a function of longitude and time. The directions of westward and eastward are indicated at the *top* of the figure. One example of the westward movements is marked by *dashed white arrow*. The example of westward movement starts from 0401:00 to 0403:30 UT

Figure 3 shows the temporal evolution of the wave-like substorm arc intensity versus the longitude and the distance along the arc in the SNKQ ASI field-of-view from 0401:00 to 0405:00 UT. The arc intensity variation along the east–west direction is calculated by summing up the bright arc intensity in the latitude direction. The arc intensity evolution shows clearly not only the azimuthal variation of the arc wave-like structure but also the westward movement of the bright spots. The locations of the bright spots change with time and tend to move towards the west. The dashed white arrow indicates one of the westward movements as an example. Several westward movements of the bright spots similar to the example can also be identified in Fig. 3. During 0401:00–0403:50 UT, the westward propagation speed of the bright spots varies from ~0.5 to ~3.5 km/s for weak to strong bright spots, and the average moving speed is ~2 km/s. However, during 0403:50–0404:30 UT, the westward propagation speed of the brightest spot is ~2 km/s.

To determine the azimuthal mode structure of the wave-like substorm arc, we perform the Hilbert-Huang Transformation (HHT) analysis (Huang et al. 1998, 1999, 2003, 2009; Wu and Huang 2004, 2009; Chang et al. 2012) and obtain the azimuthal mode number spectrum of the arc intensity structure. The HHT method has advantages in computing the so-called nonlinear “instantaneous wave frequency/number” from the temporal/spatial distribution of data. To analyze the auroral arc wave-like structure, the HHT analysis is first applied to decompose the azimuthal arc intensity variation along the arc into intrinsic mode functions (IMFs) and the trend. These IMFs are oscillatory functions and represent different wavelength scales along the arc. The trend represents the average arc intensity along the arc. Then, the local amplitude and wave number (~*A*(*x*)*e*^*ik*(*x*)*x*^ + c.c. in the local WKB sense) are constructed for each intrinsic mode function along the azimuthal arc distance *x*. Because the local amplitude and wave number vary along the arc distance *x*, we can treat the local amplitude as a two-dimensional function of the local wave number and the distance *x* along the arc. The square of the local wave amplitude and the wave number are then averaged over the arc length to obtain the wave number spectrum of the wave amplitude square (or is called the power spectral density (PSD)). For this substorm event, the PSD is obtained by averaging the square of the instantaneous amplitude over ~10° MLON segment of the wave-like substorm arc where the wave-like arc brightening structure is prominent.

**Fig. 4:**
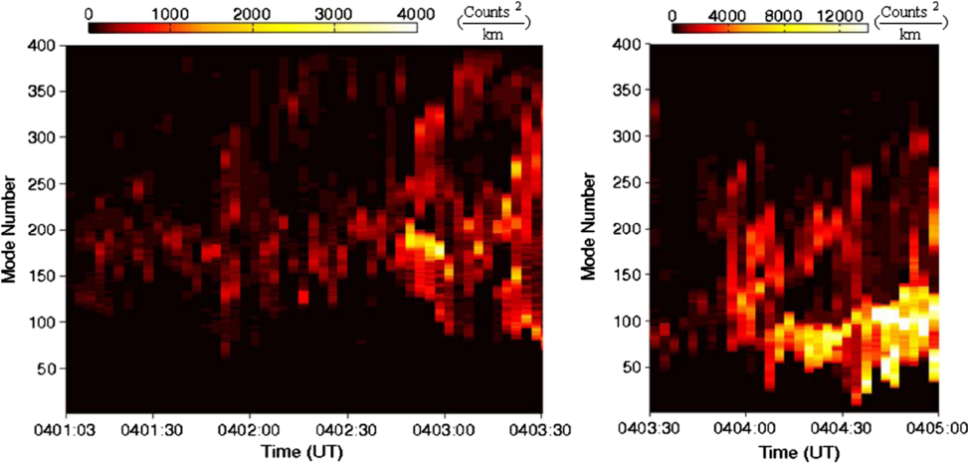
The temporal evolution of the azimuthal mode number spectrum of the phase space density (PSD) of the wave-like auroral arc intensity structure. The PSD is defined to be the square of the arc intensity averaged over the arc length. Note that the intensity color scale is different in the two panels

The temporal evolution of the azimuthal mode number spectrum of the arc intensity PSD structure during 0401–0405 UT is given in Fig. 4. Note that the intensity color scale is different in the two panels in Fig. 4. From the left panel of Fig. 4, the most significant growth of the PSD azimuthal mode number spectrum peaks at *M*~180–190 and then spreads to both higher and lower azimuthal mode number values over a wide range (*M*~120–300) during 0401:00–0402:40 UT. Then, *M*~170–190 PSD intensify during 0402:40–0403:10 UT, which coincides with the first exponential arc intensity growth as shown in Fig. 2. This may indicate that an exponentially growing instability with *M*~170–190 dominates the arc structure. During ~0403:10–0403:50 UT, the peak PSD decreases, but spreads to even higher *M* value of ~350 and lower *M* value of ~80. However, as shown in the right panel of Fig. 4, the PSD starts to intensify again from ~0403:50 UT in the *M*~80–200 range with peak at *M*~100–130. But the peak PSD spectrum shifts to lower and narrower azimuthal mode number range of *M*~60–90 during ~0404:15–0404:30 UT, which coincides with the second exponential growth of the average arc intensity during 0403:50–0404:30 UT as shown in Fig. 2. After ~0404:30 UT, the peak PSD spreads to *M*~40–130 corresponding to the substorm expansion (Rae et al. 2010; Chang et al. 2012). Thus, 0404:30 UT is considered as the auroral substorm onset time.

#### THEMIS satellite observation of the 26 Feb. 2008 dipolarization event

**Fig. 5:**
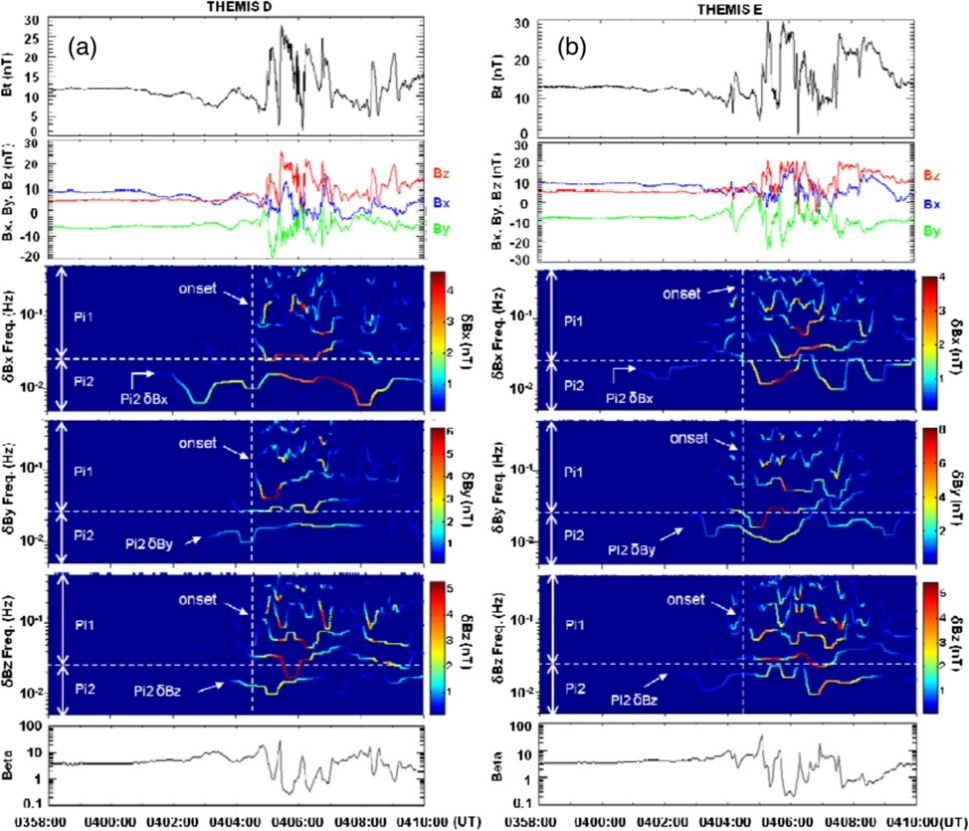
The total magnetic field *B*_*t*_ and the three magnetic field components *B*_*x*_, *B*_*y*_, *B*_*z*_ in the GSM coordinate and the amplitude–frequency–time spectrogram of the decomposed intrinsic mode functions (IMFs) obtained by the HHT method for the δ*B*_*x*_, δ*B*_*y*_, and δ*B*_*z*_ perturbations observed by (**a**) Th D and (**b**) Th E spacecraft, respectively. The *bottom* panels show the ion beta, which is much larger than unity prior to the substorm onset. Note that the Pi2 disturbances are excited prior to both the substorm onset time (~0404:30 UT) and the higher frequency disturbances. The lowest frequency IMF components are in the Pi2 frequency range

Figure 5 shows the temporal evolution of the total magnetic field *B*_*t*_ and three magnetic components *B*_*x*_, *B*_*y*_, and *B*_*z*_ in the GSM coordinates, and the amplitude–frequency–time spectrogram of the decomposed intrinsic mode functions (equivalent to different frequency components) of the perturbed δ*B*_*y*_ obtained by the HHT method from the observations as well as the ion beta observed by (a) the Th D and (b) Th E spacecraft, respectively (Chang et al. 2012). Note that the ion beta is much larger than unity prior to the substorm onset. As shown in Fig. 5, the Th D satellite observes the magnetic *B*_*z*_ dipolarization that starts at about the same time (~0404:30 UT) as the auroral expansion onset in the ionosphere. But, the Th E spacecraft observes the magnetic *B*_*z*_ dipolarization at ~0405:00 UT probably because it is located further away from the substorm initiation region in the plasma sheet. In both Th D and Th E spacecraft observations, the magnetic *B*_*z*_ dipolarization is accompanied by a significant increase in plasma flow, wave fluctuations, and high energy ion flux (Pu et al. 2010; Ogasawara et al. 2011).

As shown in Fig. 5a, the HHT analysis results of δ*B*_*x*_, δ*B*_*y*_, and δ*B*_*z*_ observed by Th D show that the Pi2 disturbances are exited in the plasma sheet prior to the auroral substorm expansion onset time of ~0404:30 UT (indicated by white dashed line), and the higher frequency Pi1 disturbances started about tens of seconds later. The Pi2 disturbances are also excited prior to the Pi1 disturbances in the Th E data shown in Fig. 5b. Similar results are found in many previous studies as well (e.g., Takahashi et al. 1987; Roux et al. 1991; Cheng and Lui 1998; Shiokawa et al. 2005; Saito et al. 2008). Because the excitation of the Pi2 disturbances precedes the onset of substorm and the Pi1 disturbances, we suggest that the Pi2 disturbances, rather than the Pi1 disturbances, are related to the cause of substorm arc formation and intensification during the pre-onset time.

**Fig. 6:**
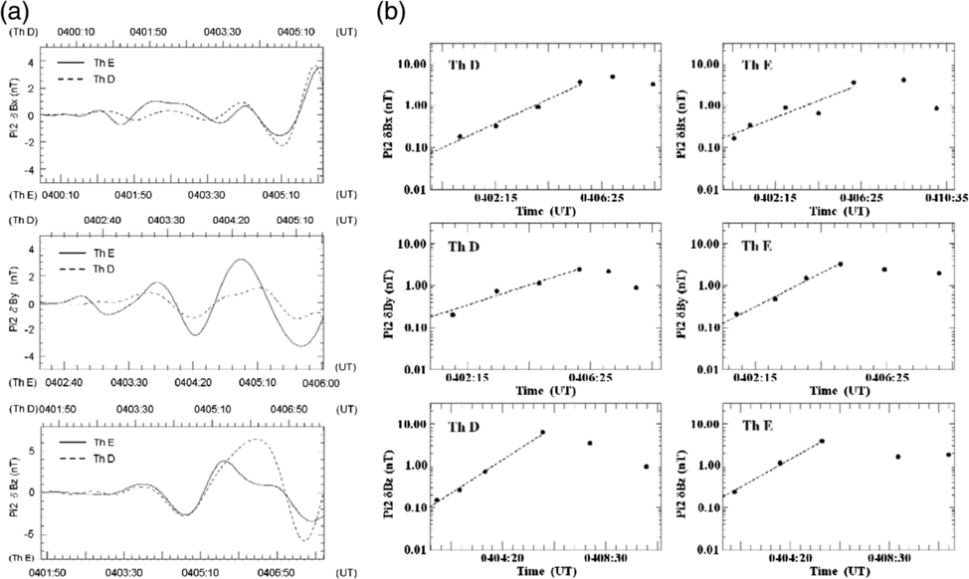
**a** The cross-phase correlation analysis for the decomposed Pi2 perturbations obtained by the HHT analysis for δ*B*_*x*_, δ*B*_*y*_, and δ*B*_*z*_ perturbations observed by Th D and Th E spacecraft, respectively. From the time series plots of the two Pi2 perturbations, the delay time is ~21 s for the Pi2 δ*B*_*x*_ disturbance (correlation coefficient is 0.79), ~28 s for the Pi2 δ*B*_*y*_ disturbance (correlation coefficient is 0.74), and ~16 s for the Pi2 δ*B*_*z*_ disturbance (correlation coefficient is 0.76) from the Th D to Th E spacecraft. **b** The exponential growth behavior of the peak amplitude of the Pi2 δ*B*_*x*_, δ*B*_*y*_, and δ*B*_*z*_ perturbations observed by Th D and Th E spacecraft

Because Pi2 disturbances were observed by both Th D and Th E spacecraft, we performed the cross-phase correlation analysis for the decomposed Pi2 δ*B*_*x*_, Pi2 δ*B*_*y*_, and Pi2 δ*B*_*z*_ data obtained by the Th D and Th E spacecraft in a time interval of ~3.5 min around the substorm onset time of 0404:30 UT. Figure 6a shows the temporal evolution of Pi2 δ*B*_*x*_, Pi2 δ*B*_*y*_, and Pi2 δ*B*_*z*_ observed by the Th D and Th E spacecraft. Note that the time scale of Pi2 δ*B*_*x*_, Pi2 δ*B*_*y*_, and Pi2 δ*B*_*z*_ perturbations observed by Th D (Th E) is shown in the top (bottom) horizontal scale. We note that the amplitude of the Pi2 disturbances shows exponential growth prior to the onset of auroral arc expansion (Fig. 6b). The magnetic perturbations observed by the Th D and Th E spacecraft are well correlated. The correlation coefficients are largest if the disturbances propagated westward from the Th D location to the Th E location with a delay time (*t*_d_) of 21, 28, and 16 s for Pi2 δ*B*_*x*_, Pi2 δ*B*_*y*,_ and Pi2 δ*B*_*z*,_ respectively.

The correlation coefficient between these two Pi2 δ*B*_*y*_ perturbations shown in Fig. 6a is ~0.735. The Th D and Th E spacecraft are located at (−10.6, 4.2, −1.9) R_E_ and (−9.8, 4.8, −1.6) R_E_ in the GSM coordinates, and the azimuthal separation distance between Th D and Th E spacecraft is ~1 R_E_. By assuming that the disturbance propagated westward in a distance of less than one wavelength, the westward propagation velocity is estimated to be *V*_p_ = Δ*R*/*t*_d_~240 km/s. The period *T*_0_ is ~60 s from Fig. 6a, then the wavelength is *λ* = *V*_p_*T*_0_ = 14,400 km~2.25 R_E_, and the azimuthal mode number is *M* = 2*πR*/λ = 32 for *R* = 11.5 R_E_, which is not consistent with the azimuthal mode number spectrum of the auroral wave-like arc shown in Fig. 4. However, the Pi2 disturbance may have propagated more than one wavelength, then the delay time should be *t*_d_ + *nT*_0_, where *n* is the number of wavelength (*λ*) the Pi2 disturbance has propagated. If we choose *n* = 2, then the westward propagation phase velocity is *V*_p_ = Δ*R*/(*t*_d_ + 2*T*_0_)~45 km/s. Then *λ* = *V*_p_*T*_0_~2700 km~0.42 R_E_ and the azimuthal mode number is *M* = 2*πR*/*λ*~172 (for *R* = 11.5 R_E_), which is consistent with the wave-like arc’s azimuthal mode number spectrum of *M*~170–190 during 0401:00–0403:30 UT estimated from Fig. 4. Because the separation distance between the mapped footprints of Th D and Th E spacecraft is ~156 km, the Pi2 disturbance phase velocity mapped to the ionosphere is ~1.8 km/s, which is also close to the wave-like arc bright spot propagation velocity of ~2 km/s during 0402:00–0404:30 UT.

It is to be commented that by choosing that the Pi2 disturbance has propagated from the Th D spacecraft location for more than two wavelengths before reaching the Th E spacecraft location, there must be two corresponding bright spots between the Th D and Th E fieldline footprints, which is also observed in the auroral arc structure before ~0404:00 UT shown in Fig. 1. This is consistent with the proposal that the arc bright spots are produced by electrons accelerated by the Pi2 wave parallel electric field downward into the ionosphere because the azimuthal structure of the Pi2 parallel electric field between the Th D and Th E spacecraft must have two upward field-aligned pointing region, which can accelerate electrons into the ionosphere to produce the arc bright spots. Thus, the choice of *n* value can be re-examined by checking the number of observed auroral bright spots.

Similar analysis has also been performed for Pi2 δ*B*_*x*_ and Pi2 δ*B*_*z*_ perturbations, and the results are similar to the case for Pi2 δ*B*_*y*_ for the 26 Feb. 2008 substorm event. However, not all magnetic components have similar properties for other substorm events. Thus, we investigate the correlation between the wave-like substorm arcs and Pi2 disturbances of different magnetic components for major substorm events observed by THEMIS spacecraft during 2008–2009 in “Correlation between substorm wave-like arcs and Pi2 disturbances in near-earth plasma sheet”.

## Correlation between ionospheric and magnetospheric features of major substorm events during 2008–2009

Next, we investigate the correlation between the auroral wave-like arc structure and magnetospheric Pi2 disturbance features for the major substorm events observed by the THEMIS spacecraft and GBO. During the campaigns of THEMIS spacecraft major tail conjunctions during 2008–2009, the apogees of the Th D and Th E spacecraft are located in the near-Earth plasma sheet (at *X*~−10 R_E_) with spatial separation roughly in the azimuthal direction when the spacecraft are cruising the night-side magnetosphere. During 2008–2009, THEMIS spacecraft observed 62 major substorm dipolarization events as described in the substorm timing table provided by UCLA (http://www.igpp.ucla.edu/themis/events/). From these 62 major substorm dipolarization events, we could identify only 37 auroral substorm events which are characterized by the auroral arc intensification followed by the auroral expansion/breakup within a few minutes prior to the substorm expansion onset using the THEMIS GBO network. In the other 25 events, it is difficult to identify the characteristic timing of the auroral substorms due to either the lack of explicit auroral expansion morphology or cloudy skies over the auroral observation area, etc.

The onset of auroral arc expansion/breakup is considered as the auroral substorm onset in this study. Among the 37 auroral substorm events, we further identify 31 events (~83 % of the events) that show the intensification of the wave-like arc structure for tens of seconds prior to the substorm onset (Voronkov et al. 1999; Donovan et al. 2008; Liang et al. 2007, 2008; Uritsky et al. 2009; Sakaguchi et al. 2009; Henderson 2009; Rae et al. 2009a, b, 2010; Chang et al. 2012). The wave-like arc features are azimuthally spaced bright spot structures that move along the arc direction prior to the auroral expansion onset. These 31 auroral substorm events with clear wave-like arc features are analyzed to provide information of the characteristic azimuthal mode structure and movement of the substorm onset arc. In the other 6 auroral substorm events (~17 % of the events), we could not find clearly the feature of wave-like arc brightness structure prior to the auroral substorm expansion. Below, we will present the analysis results of both the ionospheric wave-like arc features and the corresponding magnetospheric Pi2 features for these 31 substorm events.

### Magnetic latitude dependence of wave-like arc structure

**Fig. 7:**
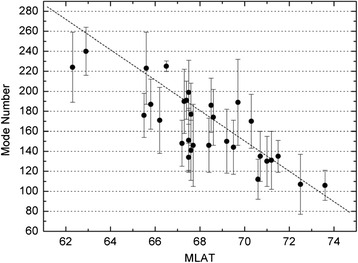
The dependence of the recursive mean of the azimuthal mode number on the arc MLAT position for the 31 wave-like substorm auroral arc events. The recursive mean of the azimuthal mode number is computed from the arc PSD histogram shown in Fig. 4 by averaging over 60 s before the azimuthal mode number values spreads to a wide range. The recursive mean is regarded as the mean value of the azimuthal mode number and the error bar represents the ±*σ* deviation from the mean value

First, we show that the substorm wave-like arc structure depends on the arc magnetic latitude (MLAT) location. We study the arc azimuthal mode spectrum for all the 31 substorm wave-like arc events by using the HHT analysis described in “Auroral arc features of the 26 Feb. 2008 substorm”. For each of the 31 substorm wave-like arc events, the recursive mean of the azimuthal mode number spectrum is computed from the arc PSD histogram, such as that shown in Fig. 4, by averaging over 60 s before the auroral expansion onset and before the azimuthal mode number (*M*) spectrum spreads to a wide *M* range. The recursive mean is regarded as the mean azimuthal mode number value of the azimuthal mode number spectrum. We also compute the standard deviation *σ* of the azimuthal mode number spectrum. Figure 7 shows the recursive mean of the azimuthal mode number spectrum and the ±*σ* deviation (the error bar) from the recursive mean value versus the MLAT of the arc location for these 31 substorm wave-like arc events. It is found that the recursive mean azimuthal mode number value of the wave-like arc brightness structure is inversely related with the arc MLAT location with a correlation coefficient of ~0.81. The mean azimuthal mode number value ranges from ~100 to 240. This feature provides the constraint on the possible theory/model of substorm onset arc formation mechanism.

### Correlation between substorm wave-like arcs and Pi2 disturbances in near-earth plasma sheet

Because Pi2 disturbances often precede the magnetic dipolarization onset in the plasma sheet as well as the substorm auroral expansion onset in the ionosphere, we investigate the correlation between the Pi2 disturbances in the near-Earth plasma sheet and the corresponding substorm wave-like arcs in the ionosphere around the substorm onset time. To perform the correlation study, we search for substorm events observed by both the Th D and Th E spacecraft with the requirements that (1) the wave-like substorm arc features persist for at least 20 s prior to auroral expansion/breakup, (2) both the Th D and Th E spacecraft observe Pi2 disturbances prior to magnetic dipolarization in the near-Earth plasma sheet, (3) the auroral substorm onset occurs at about the same time (within ~30 s) as the magnetic dipolarization in the plasma sheet, and (4) the ionospheric footprints of Th D and Th E mapped by using the T96 model (Tsyganenko 1995) are close to the wave-like substorm arc. For the 31 wave-like substorm arc events, in the Th D and Th E spacecraft observations, we find only 10 dipolarization events that satisfy all the event selection criteria. Table 1 lists these 10 substorm dipolarization events together with the features of the corresponding wave-like substorm arcs.

**Table 1:** Ten substorm dipolarization events in the plasma sheet that have accompanying wave-like arc brightening structure (WLABS) in the ionosphere

Event (yyyy/mm/dd)	Initial brightening of the WLABS (UT)	Onset of auroral expansion (UT)	*M*_1_	Number of period	Velocity of WLABS (km/s)	Phase velocity of Pi2 δ*B*_*y*_ disturbance (km/s)	*M*_2_
2008/02/02	0739:03	0741:06	187	2	−3	−38	205
2008/02/18	0913:18	0916:36	225	2	−1.6	−44	172
2008/02/26	0401:03	0404:30	185	2	2	45	172
2008/02/26	0449:00	0455:18	112	1	1.7	75	106
2008/03/05	0604:33	0607:33	134	1	2.6	68	114
2008/03/11	0533:15	0535:51	177	2	1.3	40	198
2008/03/23	0546:06	0549:39	199	2	0.93	36	214
2008/03/23	0557:09	0557:57	186	2	1.2	43	181
2009/03/19	0651:39	0654:54	177	2	−1.6	−42	185
2009/03/23	0603:51	0606:36	191	2	1.82	46	167

*M*_1_ and *M*_2_ are the recursive mean values of azimuthal mode number calculated in accordance with the wavelength defined by the WLABS and the Pi2 δ*B*_*y*_ disturbance, respectively. And *n* is the number of wavelength that the Pi2 δ*B*_*y*_ disturbance has propagated between Th D and Th E spacecraft

Positive (negative) moving velocity of wave-like arc brightening structure indicates westward (eastward) movement. Positive (negative) phase velocity of Pi2 δ*B*_*y*_ disturbance means westward propagation from Th D to Th E (eastward from Th E to Th D)

**Fig. 8:**
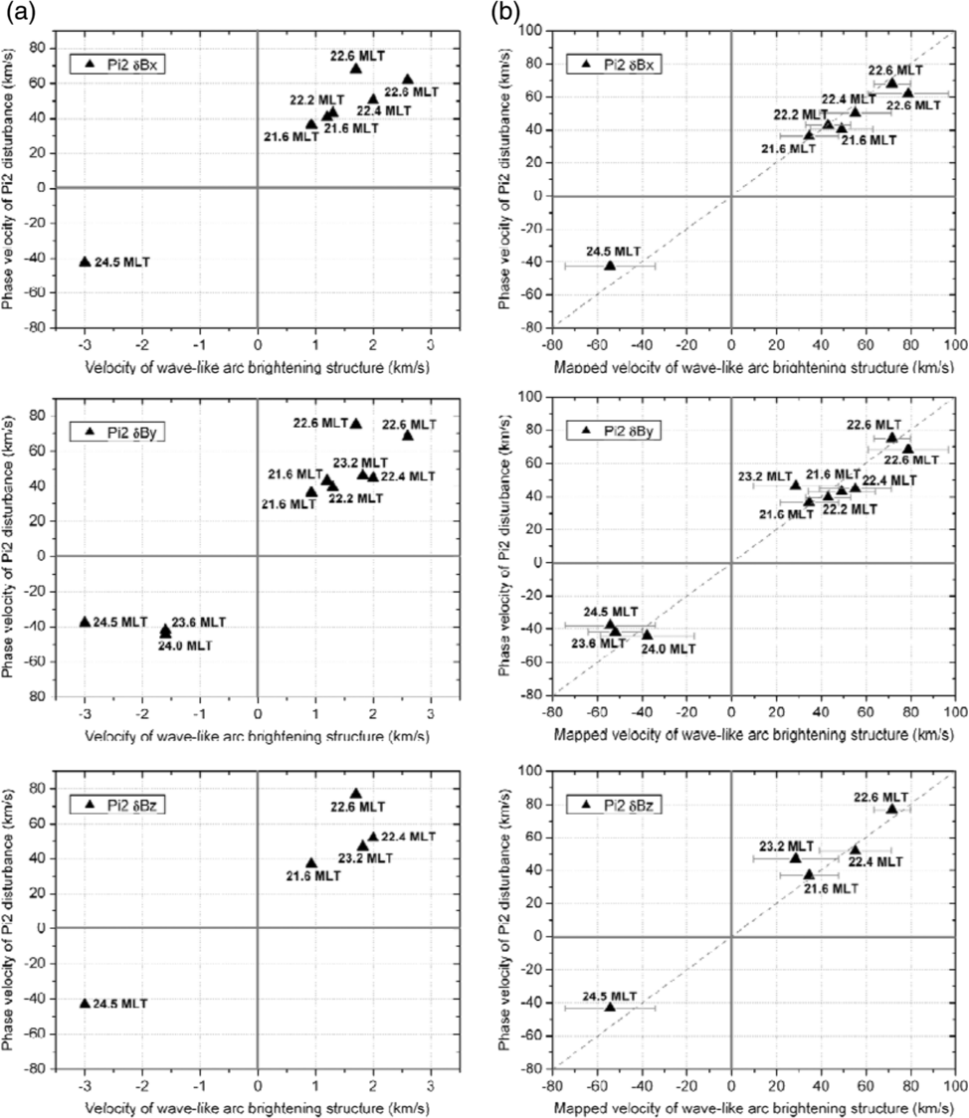
**a** For the 10 substorm events listed in Table 1, the phase velocity of Pi2 disturbance of δ*B*_*x*_, δ*B*_*y*_, and δ*B*_*z*_ observed by Th D and Th E spacecraft versus the velocity of the wave-like arc brightening structure. The positive (negative) moving velocity of wave-like arc brightening structure indicates westward (eastward) variation. The positive (negative) phase velocity of Pi2 δ*B*_*y*_ disturbance means propagation from Th D to Th E (from Th E to Th D). **b** The phase velocity of Pi2 disturbance versus the velocity of the wave-like arc brightness structure movement mapped to the equatorial position using the T96 model. We also mark the standard deviation of the data points with respect to the perfect match line (*dashed line*)

We have analyzed the correlation between the wave-like substorm arcs and the Pi2 disturbances in the plasma sheet for these 10 dipolarization events by following the analysis method described in “The 26 Feb. 2008 substorm event”. In Table 1, *M*_1_ and *M*_2_ are the values of azimuthal mode number calculated from the wave-like auroral arc brightening structure and the Pi2 δ*B*_*y*_ disturbance wavelength observed in the plasma sheet, respectively. And *n* is the number of wavelength that the Pi2 disturbance has propagated from Th D to Th E spacecraft. In Table 1 and Fig. 8, the velocity of the wave-like arc brightening structure is defined by the bright spot moving velocity observed by the ground-based THEMIS ASIs during few tens of seconds before arc expansion onset. The phase velocity of Pi2 disturbances observed by the Th D and Th E spacecraft in these substorm dipolarization events and the Pi2 disturbance azimuthal mode number *M*_2_ are estimated by following the cross-phase correlation analysis method and the calculations as described in “THEMIS satellite observation of the 26 Feb. 2008 dipolarization event”. The positive (negative) moving velocity of wave-like arc brightness structure indicates westward (eastward) motion, and the positive (negative) phase velocity of Pi2 δ*B*_*y*_ disturbance means it propagates westward from Th D to Th E spacecraft (eastward from Th E to Th D spacecraft).

Figure 8a shows the phase velocity of the Pi2 δ*B*_*x*_, δ*B*_*y*_, and δ*B*_*z*_ disturbances observed by Th D and Th E spacecraft versus the moving velocity of the wave-like arc brightness structure observed prior to the substorm onset for all the 10 substorm events listed in Table 1. The MLT locations of the spacecraft are also indicated in the figure. In the analysis of the satellite magnetic field data in the plasma sheet region, it is usually difficult to separate spatial variation from the temporal variation, in particular, when the plasma sheet structure moves rapidly (such as tail flapping). Here we limit the magnetic data before and around the substorm onset time for about two wave periods of Pi2 disturbances, during which the satellite moves only a distance of much less than 1 R_E_, and we hope to isolate the temporal variation from the spatial variation of the magnetic field data. We compare the frequencies of the same Pi2 magnetic component observed by two neighboring spacecraft, i.e., Th D and Th E spacecraft in the substorm events presented in Fig. 8a. If the frequencies of the Pi2 magnetic component observed by the two neighboring spacecraft before and around the auroral expansion onset time are different by a factor of 1.5 or larger, they are considered to be different waves. However, if the difference in the frequencies is less than a factor of 1.5, we can consider that these two observed Pi2 magnetic disturbances may be related to the same/similar wave. For different waves, we can exclude them from the Pi2 phase velocity calculation, and they are not included in the correlation study results presented in Fig. 8a.

Next, we map the movement of the wave-like arc brightness structure to the equatorial plane. Figure 8b shows the data points of the computed phase velocity of Pi2 disturbance versus the velocity of the wave-like arc brightness structure movement mapped to the equatorial position using the T96 model. We also calculate the standard deviation of the data points with respect to the perfect match line (dashed line in Fig. 8b) that the Pi2 disturbance propagation phase velocity equals the velocity of the wave-like arc brightness structure movement mapped to the equatorial position using the T96 model. The difference between each data point and the dashed line show the degree of correlation between the Pi2 disturbance and the auroral arc. If the difference for the individual data point is more than two times the standard deviation (>2*σ*), we can consider that the correlation is poor. From Fig. 8b, it shows that the phase velocity of the Pi2 δ*B*_*y*_ disturbances is almost linearly correlated with the moving velocity of the wave-like arc brightness structure. Of all the 10 substorm events, the Pi2 δ*B*_*y*_ disturbances and the wave-like arc brightness structure are well correlated. However, the movement velocity of the wave-like arc brightness structure has good correlation with the phase velocity of the Pi2 δ*B*_*x*_ and δ*B*_*z*_ disturbances for 7 and 5 substorm events, respectively. Thus, we consider that the Pi2 δ*B*_*z*_ do not correlate very well with the observed azimuthal movement of the wave-like arc brightness structure. Because δ*B*_*y*_ is mainly perpendicular to the ambient magnetic field, the wave-like arc structure is caused by transverse type Pi2 waves which have parallel electric field to accelerate electrons into the ionosphere. On the other hand, the Pi2 δ*B*_*x*_ and δ*B*_*z*_ may also be contributed by the compressional type waves which have perturbations in the component parallel to the ambient magnetic field. However, when the spacecraft are not located around midnight, the azimuthal propagating Pi2 disturbance should consider both δ*B*_*y*_ and δ*B*_*x*_ components in the correlation study.

## Results and discussion

In this study, we have investigated the major substorm events during 2008–2009 which are listed in the substorm timing table provided by the UCLA. In particular, we have studied the auroral substorm wave-like arc brightness features observed by the THEMIS ASIs before the auroral expansion onset and the Pi2 magnetic disturbances in the near-earth plasma sheet by THEMIS spacecraft prior to substorm dipolarization onset. We have also performed the correlation analysis of these two phenomena. Below we summarize the main findings and results of this work and discuss possible explanations.

Firstly, we have found that most of the auroral substorm events (~83 %) observed by THEMIS all-sky imagers during 2008–2009 show wave-like arc brightness structures along the substorm onset arcs with exponentially growing intensification prior to the auroral substorm expansion onset. Thus, the wave-like auroral brightening feature is commonly observed prior to the auroral substorm expansion onset. This wave-like auroral arc structure which shows exponential growth in luminosity implies the existence of instabilities in the near-Earth plasma sheet. Thus, the auroral observations give potential constraints on the instabilities that may cause the production of the substorm arcs. In Fig. 7, the mean azimuthal mode number of the substorm wave-like arc structure is in the range of ~100–240 prior to the expansion onset. Because the most unstable kinetic ballooning instability in the plasma sheet has high azimuthal mode number and low frequency in the Pi2 range, it is a candidate to explain the Pi2 disturbance observed in the plasma sheet.

In the paper by Keiling (2012), they assume a phase velocity of 150 km/s for the 60-s Pi2 wave and obtain a wavelength of 9000 km in the equatorial plane, which corresponds to the azimuthal mode number of *M* = 40. It is also noted that the *M* = 40 value obtained by Keiling (2012) was estimated for the period after the substorm onset. The *M* value difference with our results basically comes from the assumption of phase velocity. Our results are based on the auroral arc wave-like structure (“THEMIS satellite observation of the 26 Feb. 2008 dipolarization event”) and the azimuthal phase velocities of the corresponding Pi2 waves in the plasma sheet are tens of kilometers per second. It is also to be noted that the *M* = 40 value obtained by Keiling (2012) was estimated for the period after substorm onset and it is similar to our analysis result of the 26 Feb. 2008 event shown in Fig. 4, which shows that the peak *M* values are higher (~180) in the early time of arc intensification (pre-onset time), and then after a few minutes (in the expansion phase) the peak *M* values significantly become lowered to 40–100. Similar results were also reported by Pu et al. (2010).

Secondly, we found that the mean azimuthal mode number of the wave-like substorm arcs is inversely related with the onset arc geomagnetic latitude location as shown in Fig. 7, which is still yet to be understood. If we consider the ballooning instability in the plasma sheet to be the driver of the substorm wave-like arc in the ionosphere, then the magnetic latitude of the arc location can be mapped to a radial distance *R* from the earth where the ballooning instability is located. Because the azimuthal wavelength of the most unstable mode is typically on the order of ion gyroradii, *k*_ϕ_*ρ*_i_~1, where *k*_ϕ_ = *M*/*R* is the azimuthal wave number, *M* is the azimuthal mode number and *ρ*_i_ is the ion gyroradius. Thus, *M* is proportional to *RB*/*v*_i_~*R*(*n*_i_/*β*_i_)^1/2^ where *v*_i_ is the ion thermal velocity, *n*_i_ is the ion density, and *β*_i_ is the ion plasma beta value. Typically *R*(*n*_i_/*β*_i_)^1/2^ decreases as *R* increases in the range of 10 R_E_ < *R* < 20 R_E_, and thus the kinetic ballooning instability can qualitatively explain the magnetic latitude dependence of the wave-like arc azimuthal mode number.

Thirdly, the excitation of Pi2 disturbances are observed by the THEMIS spacecraft in the near-Earth plasma sheet and are found to precede both the onset of magnetic field dipolarization and the initiation of the higher frequency disturbances (in the Pi1 range). The higher frequency Pi1 disturbances are often excited tens of seconds later. The amplitude of the Pi2 disturbances also shows exponential growth prior to the onset of auroral arc expansion (Fig. 6b). The Pi2 magnetic perturbations observed by two neighboring THEMIS spacecraft are found to be well correlated for all three magnetic components, and we are able to estimate the propagation direction and phase velocity of the Pi2 magnetic perturbations as well as their azimuthal mode number. The features of the Pi2 disturbances are also consistent with the prediction of the kinetic ballooning instability.

Fourthly, we have performed correlation studies of the substorm wave-like onset arc structures observed by the THEMIS ASIs with the Pi2 disturbances observed by the THEMIS spacecraft in the near-earth plasma sheet. Both the wave-like arc bright spots and the Pi2 disturbances propagate in the azimuthal direction, either westward or eastward. From the THEMIS spacecraft observations of Pi2 disturbances and THEMIS ASI observations of substorm onset arcs during the period of 2008–2009, we have identified only 10 substorm events (listed in Table 1) that satisfy the selection criteria described in “Correlation between substorm wave-like arcs and Pi2 disturbances in near-earth plasma sheet”. We found that the mapped bright spot movement velocity of the wave-like arc structure is correlated with the phase velocity of the Pi2 δ*B*_*y*_ disturbances observed by THEMIS spacecraft in the near-Earth plasma sheet region as shown in Fig. 8b. Moreover, the mean azimuthal mode number of the wave-like auroral arc structure is also consistent with the estimated azimuthal mode number of the Pi2 δ*B*_*y*_ disturbances observed in the near-earth plasma sheet as shown in Table 1. Furthermore, because both the wave-like auroral arc and the Pi2 δ*B*_*y*_ disturbances observed in the near-earth plasma sheet show exponential growth behavior (Fig. 6b) prior to the substorm onset, it is reasonable to argue that both phenomena are produced by a common instability mechanism, and we suggest the kinetic ballooning instability as the candidate instability.

Panov et al. (2012) reported that the δ*B*_*x*_ oscillation is typical of the kinetic ballooning/interchange instability in the stretched parts of magnetotail. From our analysis results, we suggest that the movement of the wave-like arc structure is better related with the Pi2 δ*B*_*y*_ disturbances as shown in Fig. 8b. However, when the spacecraft locations are not close to midnight region, both δ*B*_*y*_ and δ*B*_*x*_ must be taken into account to describe the Pi2 disturbance azimuthal perturbation.

Finally, from the amplitude–frequency–time spectrum (Fig. 5) obtained by the HHT analysis of the THEMIS spacecraft observation of magnetic field data, the Pi2 disturbances are first excited and the Pi2 amplitude grows exponentially prior to substorm onset and the Pi1 disturbances are usually excited tens of seconds later, and both Pi2 and Pi1 disturbances coexist in the substorm expansion phase (Rae et al. 2010). During the expansion phase, the PSD of the substorm auroral arc is enhanced significantly in the low azimuthal mode number range as shown in Fig. 4. Thus, we suggest the observations can be explained in terms of the scenario that when the kinetic ballooning instability (Pi2 disturbance) grows to large amplitude and saturates around the substorm onset, the ion velocity distribution forms bump-on-tail velocity distribution which provides the free energy for exciting higher frequency instabilities such as the cross-tail current instability. As the higher frequency instabilities grow, they combine with the kinetic ballooning instability to form strong EM field turbulence and thus cause plasma transport and heating.

To draw a firmer conclusion about the role of kinetic ballooning instability in the substorm onset and expansion mechanism, more data and further analytical and simulation studies are needed to verify the theory. In particular, it is constructive to determine the 3D plasma and magnetic field structure of the magnetosphere in the late growth phase in both observation and theory/model. This will help determine the stability of kinetic ballooning instability in the late growth phase and its 3D wave structure and propagation and examine how electrons are accelerated into the ionosphere to produce the wave-like substorm onset arcs. However, we should caution that instability mechanisms (e.g., Lui 2004) other than the kinetic ballooning instability should also be examined for understanding the substorm onset phenomena. At present, the features of the kinetic ballooning instability seem to be able to explain the ionospheric and magnetospheric observational features of substorms. However, to resolve the substorm issues, more multiple spacecraft observations and advanced theory/modeling must be developed.

## Conclusions

In this paper, we focus on the crucial tens of seconds prior to substorm onset, we show the analysis result of the 26 Feb. 2008 substorm event and perform the statistical studies of the observations of wave-like substorm auroral arcs and substorm dipolarization events listed in the substorm timing table. From a statistical viewpoint, we find that (1) the azimuthal mode number values of the wave-like substorm arcs are found to be in the range of ~100–240 and decrease with increasing geomagnetic latitudes of the substorm auroral arc locations; (2) the movements of the wave-like arc brightness structures correlate well with the phase velocities of the Pi2 δ*B*_*y*_ disturbances in the near-Earth plasma sheet region. Tentatively, we suggest that kinetic ballooning instability is plausible in explanation of these analysis results. However, instability mechanisms other than the kinetic ballooning instability should also be examined for understanding the substorm onset phenomena. Indeed, to resolve the substorm issues, more studies towards advanced theory/modeling and multi-spacecraft conjunction observations and its correlation to substorm auroral activities are still desired.
